# Retinal Aging in 3× Tg-AD Mice Model of Alzheimer's Disease

**DOI:** 10.3389/fnagi.2022.832195

**Published:** 2022-06-16

**Authors:** Pedro Guimarães, Pedro Serranho, João Martins, Paula I. Moreira, António Francisco Ambrósio, Miguel Castelo-Branco, Rui Bernardes

**Affiliations:** ^1^Coimbra Institute for Biomedical Imaging and Translational Research (CIBIT), Institute for Nuclear Sciences Applied to Health (ICNAS), University of Coimbra, Coimbra, Portugal; ^2^Department of Sciences and Technology, Universidade Aberta, Lisbon, Portugal; ^3^Coimbra Institute for Clinical and Biomedical Research (iCBR), Faculty of Medicine (FMUC), University of Coimbra, Coimbra, Portugal; ^4^Center for Innovative Biomedicine and Biotechnology (CIBB), University of Coimbra, Coimbra, Portugal; ^5^Clinical Academic Center of Coimbra (CACC), Faculty of Medicine (FMUC), University of Coimbra, Coimbra, Portugal; ^6^Center for Neuroscience and Cell Biology (CNC), University of Coimbra, Coimbra, Portugal

**Keywords:** aging, artificial intelligence, age-gap, Alzheimer's disease, deep learning, animal model, retina, optical coherence tomography

## Abstract

The retina, as part of the central nervous system (CNS), can be the perfect target for *in vivo, in situ*, and noninvasive neuropathology diagnosis and assessment of therapeutic efficacy. It has long been established that several age-related brain changes are more pronounced in Alzheimer's disease (AD). Nevertheless, in the retina such link is still under-explored. This study investigates the differences in the aging of the CNS through the retina of 3× Tg-AD and wild-type mice. A dedicated optical coherence tomograph imaged mice's retinas for 16 months. Two neural networks were developed to model independently each group's ages and were then applied to an independent set containing images from both groups. Our analysis shows a mean absolute error of 0.875±1.1 × 10^−2^ and 1.112±1.4 × 10^−2^ months, depending on training group. Our deep learning approach appears to be a reliable retinal OCT aging marker. We show that retina aging is distinct in the two classes: the presence of the three mutated human genes in the mouse genome has an impact on the aging of the retina. For mice over 4 months-old, transgenic mice consistently present a negative retina age-gap when compared to wild-type mice, regardless of training set. This appears to contradict AD observations in the brain. However, the ‘black-box” nature of deep-learning implies that one cannot infer reasoning. We can only speculate that some healthy age-dependent neural adaptations may be altered in transgenic animals.

## 1. Introduction

We are currently under a neurological epidemic. Over a third of all Europeans suffer from some form of brain disease. In 2010, it was estimated that there were about 40–50 million people with dementia worldwide (Nichols et al., 2019). Alzheimer's disease (AD) accounts for 50–75% of all cases of dementia (Prince et al., 2016). This is a persistent, long-lasting condition that has an enormous impact on the patients' lives and their loved ones. Worst of all, these numbers are not slowing down. With improved care in many countries and increasing life expectancy, numbers are expected to climb over the coming decades. The rising prevalence and mounting economic burden poses a large and growing threat to every government in the world.

Brain imaging can be expensive, invasive, and challenging. However, the retina and the optic nerve are also part of the central nervous system (CNS), sharing their embryonic origin with the brain. There are many similarities between the eye and the brain, in their anatomy, vascularization, and mechanisms. As so, the eye is a readily available, inexpensive window to assess neuropathology. Post-mortem data shows amyloid-beta deposits in and around melanopsin retinal ganglion cells for AD subjects (La Morgia et al., 2016). Moreover, the presence of hyperphosphorylated tau in the innermost layers of the retina in mice has been demonstrated (Schön et al., 2012). Grimaldi et al. (2018) showed that amyloid-beta plaques, hyper-phosphorylated tau tangles, ganglion neuron degeneration, astrogliosis, and microglial activation were already detectable at a pre-symptomatic stage in the 3× Tg-AD mice model. Recently, it has been observed that several microRNAs were deferentially expressed in the retina of 3× Tg-AD mice when compared to age-matched wild-type (WT) mice (Burgaletto et al., 2021). Imaging methods such as optical coherence tomography (OCT) can image the retina *in vivo, in situ*, and noninvasively. AD related retinal OCT changes have already been reported in both humans and animal models of the disease (Hart et al., 2016; Harper et al., 2020).

Animal models are essential tools in the study of human pathology. These allow us to better understand the pathophysiology and guide us in the development of novel therapeutics. Triple transgenic (3× Tg-AD) mice harbor three human mutant genes: the Swedish amyloid precursor protein (APPswe), presenilin 1 (PSEN1), and microtubule-associated protein tau (MAPT), which are associated with familial AD (Oddo et al., 2003). The observed progression timeline and localization appear to mimic observations in humans. As so, this animal model has been an important tool in the study of the disease.

In this work, we take advantage of the broad spectrum of deep learning (DL) to take a global look at retinal aging in age-matched 3× Tg-AD *vs* WT mice. It has long been established that several age-related changes in the brain are more pronounced in AD (Fox and Schott, 2004; Sperling, 2007; Toepper, 2017). While the same has been theorized for the decrease in retinal thickness, results have been inconsistent, with both thickening and thinning being reported. In Ferreira et al. (2021), differences were found in all but the outer nuclear layer (ONL), including thickening of the retinal nerve fiber layer—ganglion cell layer (RNFL-GCL) complex, and thinning of all the remaining layers. Chidlow et al. (2017) found no differences in retinal layer thickness when investigating early stages in a mouse model of AD. Similarly, Song et al. (2020) found significant thinning of the RNFL only. DL has already demonstrated potential in medical image analysis in multiple fields. Here, using DL as a modeling tool, allows us to take an unrestricted approach to retinal age evaluation, not focused on layer thickness, but instead theoretically considering all the existing information from the OCT scan.

## 2. Materials and Methods

### 2.1. Ethics Statement

This study was approved by the Animal Welfare Committee of the Coimbra Institute for Clinical and Biomedical Research (iCBR), Faculty of Medicine, University of Coimbra (approval ORBEA 16/2015, addendum 6/2018). All procedures involving mice were conducted as per statement for animal use by the Association for Research in Vision and Ophthalmology, and in agreement with the European Community Directive Guidelines for the care and use of nonhuman animals for scientific purposes (2010/63/EU), transposed into the Portuguese law in 2013 (DL113/2013).

### 2.2. Data

**Figure 1:**
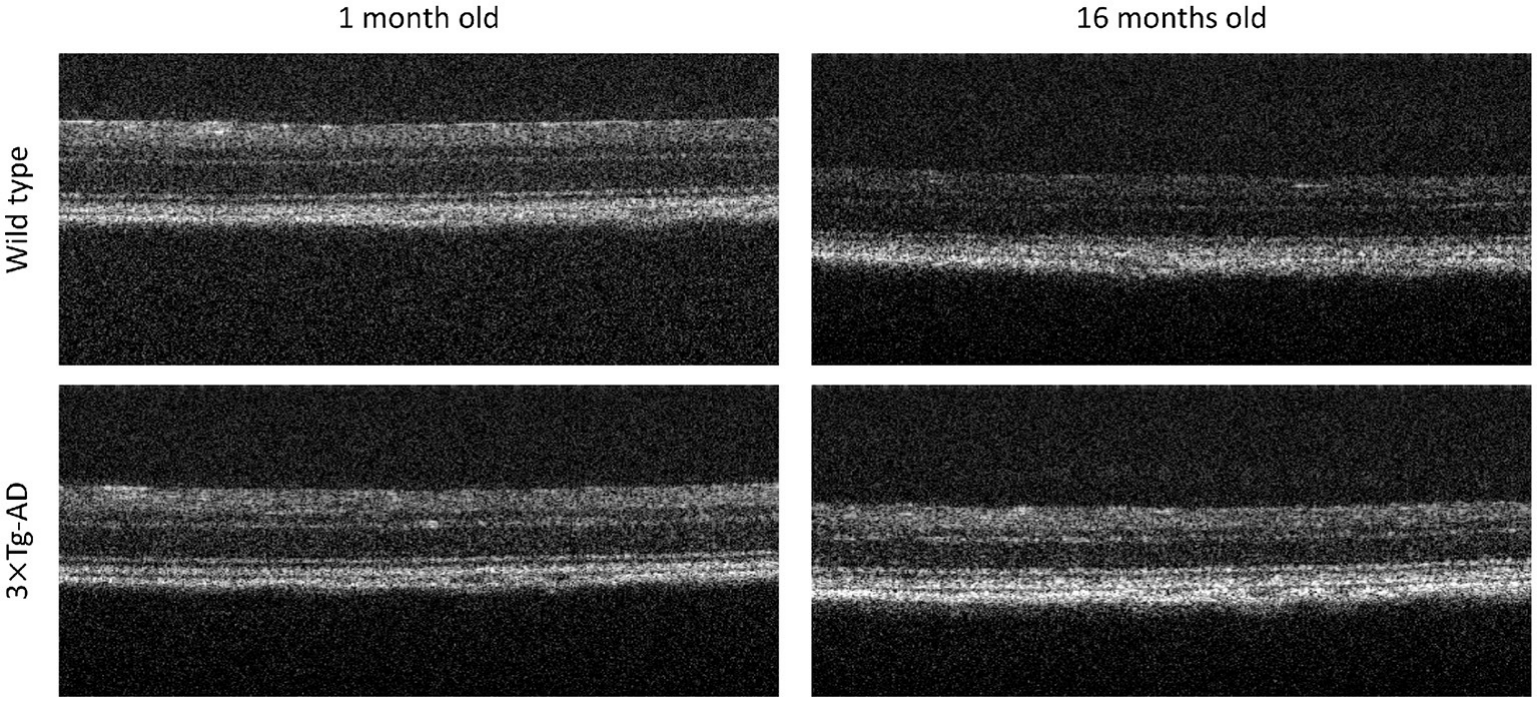
Representative optical coherence tomography B-scans. **Top** to **bottom**, B-scans of a Wild type and a triple transgenic familiar Alzheimer's disease mouse model (3× Tg-AD), imaged at 1 and 16 months old, **right** to **left**, respectively.

In total, 57 WT C57BL6/129S and 60 3× Tg-AD age-matched male mice were used. Mice were randomly split into two sets: DS1, containing 80.0% of the population, and DS2, containing the remainder 20.0%. The same ratio of transgenic mice was forced in both sets. For each mouse, volumes were acquired from both eyes at the ages of 1, 2, 3, 4, 8, 12, and 16 months old using a Micron IV OCT System (Phoenix Technology Group, Pleasanton, CA, USA), resulting in 1,444 volumes of 512 B-scans each (2D images composed of 512 one-dimensional scans over depth, see Figure 1), imaged at a predefined retinal region (directly above the optic disc). From these, regularly spaced B-scans (5 B-scans apart) were selected, totaling 14.730 B-scans (512 × 1,024 pixels). Each selected B-scan was cropped to obtain a region (512 × 512 pixels) centered at the retina whose location was automatically determined. Each B-scan is normalized to have zero mean, and unit-variance.

All mice were housed and maintained on a 12 h light/dark cycle with free access to food and water at the vivarium of the Coimbra Institute for Clinical and Biomedical Research (iCBR), Faculty of Medicine, University of Coimbra.

Before OCT acquisition, each mice was anesthetized using a mixture of 80 mg/kg of ketamine (Nimatek; Dechra) and 5 mg/kg xylazine (Sedaxylan; Dechra). Mice pupils were dilated using a solution of 0.5% tropicamide (Tropicil; Edol) and 2.5% phenylephrine (Davinefrina; Dávi). Additionally, oxibuprocaine (Anestocil; Edol), a local anesthetic, was used. Eyes were regularly lubricated using eye drops (1% carmellose: Celluvisc; Allergan).

The OCT system used has an imaging depth of 1.4 mm and axial resolution of 3 μm, as determined by the bandwidth and central wavelength, respectively 160 and 830 nm, of the superluminescent diode used. All acquisitions were performed by the same operator. To guarantee a well spread-out B-scan selection, some limitations were imposed on the random process: minimum separation between B-scans and minimum number of selected B-scans per volume. Detailed data characterization can be found in the Supplementary Material. B-scans are saved as non-compressed TIFF file images.

### 2.3. Age Modeling

Deep-learning is not limited to any pre-existing knowledge. Instead, layer-to-layer more and more complex representations are created, and theoretically, no abstract representation is off limits. As so, it is our tool of choice to model and compare the retinal aging in both groups.

**Figure 2:**
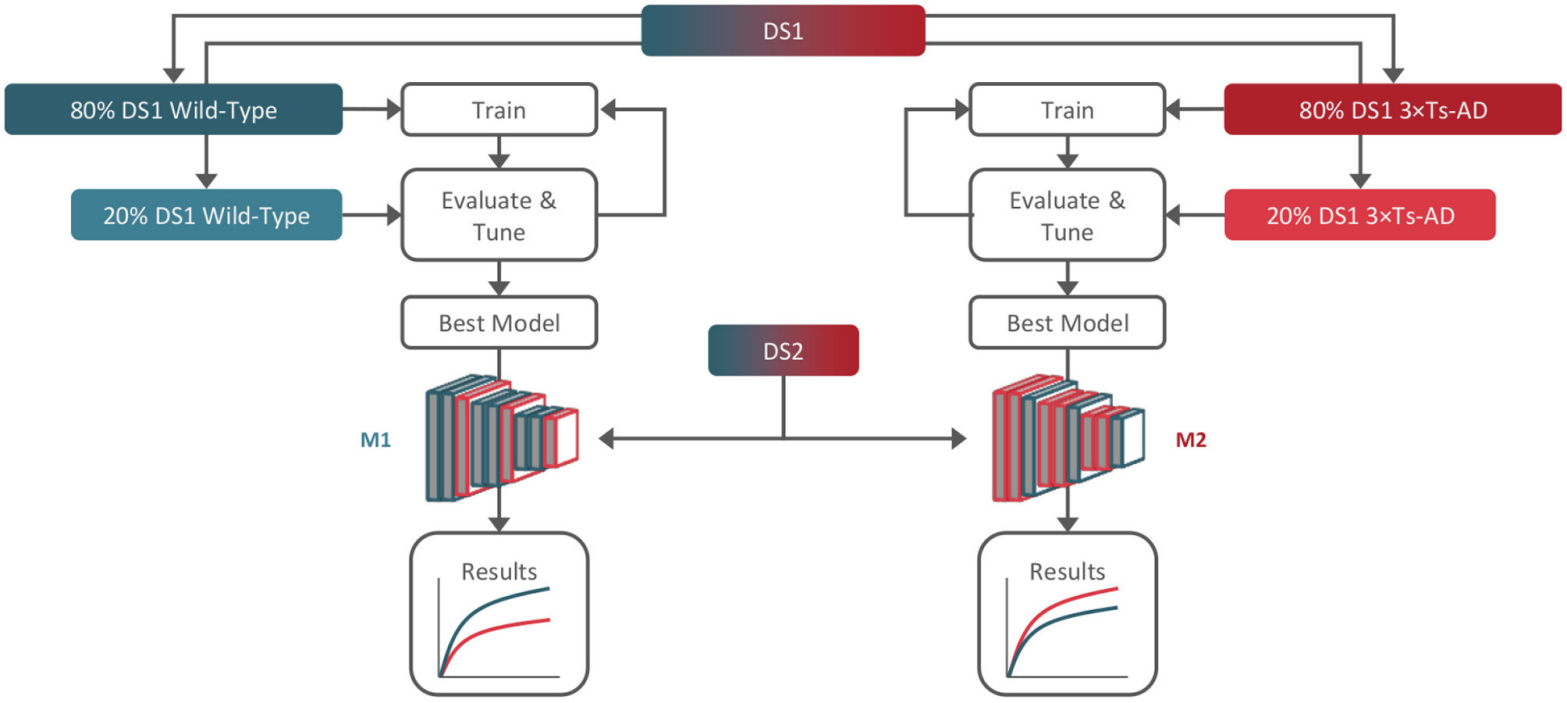
Training, tuning, and testing workflow. Dataset 1 (DS1) was used for training and tuning (80/20%) of two models, M1 and M2, using only wild-type and 3× Tg-AD B-scans, respectively. Dataset 2 (DS2) containing both genotypes was used for hold-out testing with both models.

We trained and tuned two different models, each trained with only data from one group: one using only B-scans from DS1 WTs (M1), and the other using only B-scans from DS1 transgenic mice (M2). Both models were then used to predict age in DS2 B-scans, which included B-scans from both WT and transgenic mice. This setup allows us to evaluate the consistency of our results and eliminate training bias. Figure 2 summarizes the study workflow.

We used transfer-learning, dropout regularization, and artificial data augmentation as overfitting mitigation tools. The base network of choice was a DenseNet (Huang et al., 2017) pre-trained on ImageNet (Deng et al., 2009). Data augmentation (rotation, scaling, and horizontal reflection) was applied to training only. For each model, 20.0% of the training images were used for hyperparameter tuning.

Age prediction is achieved using the best performing hyperparameter combination as assessed independently for each model in the respective tuning set (grid-search selecting dropout, learning-rate, momentum, and training steps). For uncertainty estimation, variational dropout was used (Gal and Ghahramani, 2016), i.e., 50 different prediction calls are made per image using dropout regularization to act as the response of a group of various models that can be interpreted as a Bayesian probability distribution. Final prediction is computed as the average of the 50 calls, and uncertainty as its standard deviation.

### 2.4. Statistics

Kolmogorov–Smirnov test was used to assess normality of age distribution per group, at each time-point. Statistical differences between groups (WT vs. 3× Tg-AD) were tested using the non-parametric Mann–Whitney test, since most of the normality tests were statistically significant. All tests were performed with the IBM SPSS 27 software.

## 3. Results

**Figure 3:**
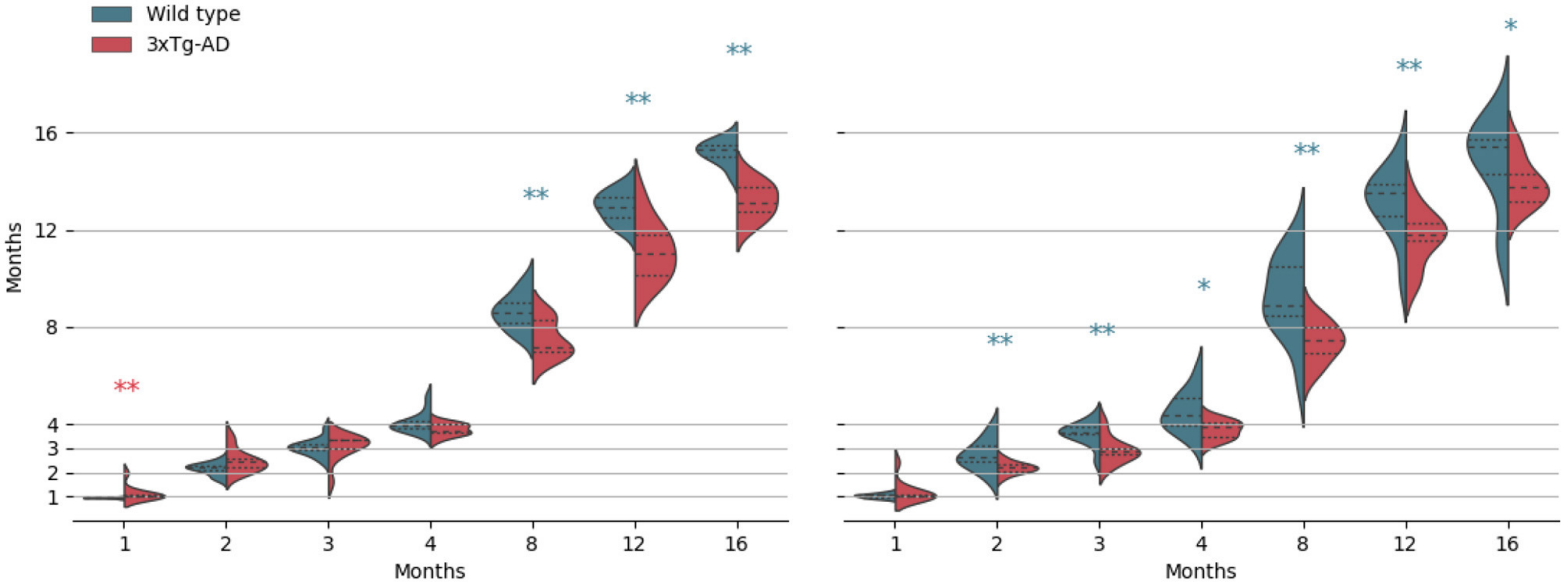
Kernel density estimates of the predicted age, separated per class (wild-type vs. 3× Tg-AD), for each acquisition time-point. From left to right, results for the wild-type and the 3× Tg-AD trained models. Median (dashed), and first and third quartiles are shown. *p < 0.05, **p < 0.01. Asterisk color indicates a higher median value.

Kernel density estimates for each acquisition time-point, separated per class, are shown in Figure 3, for both M1 and M2 models (WT and 3× Tg-AD trained, respectively). Overall, independently of training type, it was possible to predict mice age with a reasonable degree of precision, achieving a mean absolute error (MAE) of 0.875±1.1 × 10^−2^ and 1.112±1.4 × 10^−2^ months, for M1 and M2 predictions, respectively. WT training achieved slightly better performance. As expected, in both cases, prediction in the trained class was better than the opposite, MAE of 0.739±1.6 × 10^−2^ (WT) vs. 1.019±1.5 × 10^−2^ (AD) months for M1, and MAE of 0.901±1.7 × 10^−2^ (AD) vs. 1.310±1.8 × 10^−2^ (WT) months for M2.

As shown, there is a retinal age gap. There are differences in retinal aging between the two classes for both training conditions. However, contrarily to what we expected, after month 4, WT retinas appear to be consistently predicted as older than 3× Tg-AD retinas. Exact *p*-values of the Kolmogorov–Smirnov and Mann–Whitney tests can be found in the Supplementary Material.

## 4. Discussion

Even with years of research, AD timely diagnosis is still lacking. Despite the considerable advantages of imaging methods such as magnetic resonance imaging (MRI) and positron emission tomography, these are not suitable as screening methods due to their cost and lack of generalized availability. The retina offers the incredible opportunity to directly observe the impact of AD on neuronal tissue (Chiquita et al., 2019), rendering it attractive. Nevertheless, this field of research is still underdeveloped. Most approaches so far have looked at layer thickness, having gotten mixed results, with both thickening, thinning, and no differences being reported for individual retinal layers (Chidlow et al., 2017; Song et al., 2020; Ferreira et al., 2021). While important, thickness analysis forgoes a wealth of information that can be captured by OCT.

In this work, we evaluated how the aging of the retina is affected in an animal model of AD. Animal models were a natural choice, since matching pathology duration was mandatory, and medical data is often difficult to gather in the quantities needed for effective DL utilization. These models have been of pivotal importance in the study of AD. Nevertheless, further studies with human data will be required to check whether the findings in humans are like those obtained here.

We modeled the aging of the mouse retina and its aging when a pathological condition affects the CNS, and used an independent dataset to test the two resulting models. Because each model was trained with a different group (WT or 3× Tg-AD), we are able to eliminate training bias.

We were able to predict the age of the retina with a low average error independently of training on WT or 3× Tg-AD B-scans. Thus, DL was used to create a reliable retinal OCT aging marker. We have also shown that retina aging is distinct in the two classes. Our results clearly indicate that the presence of the three mutated human genes in the mouse genome has an impact on the aging of the retina. However, our results also suggest that, in some sense, there is a complex underlying biological cause resulting in age-dependent alterations. Unexpectedly, after month 4, retinas from wild-type mice appear to be consistently predicted as older than retinas from transgenic mice, independently of the training group. This appears to contradict what has been observed for AD, where some age-related changes such as, neural tissue thinning, neural activity and functional connectivity impairments were shown to be more pronounced in AD (Fox and Schott, 2004; Sperling, 2007; Toepper, 2017; Chiquita et al., 2019). It also appears to contradict findings in Li et al. (2017) and Löwe et al. (2016), where AD patients presented a positive brain-age gap (greater brain aging), and findings in Gaser et al. (2013), where brain-predicted age was a significant predictor of dementia progression within 3 years of baseline MRI scan.

In prior studies using the same 3× Tg-AD strain, unpublished results from our research group failed to identify differences in neuroinflammatory markers and neural cell death, among others, rendering findings herein even more interesting. It was previously shown that aging affects differently the gene expression in male and female mice brains (Zhao et al., 2016). Male mice presented significant brain alterations at older ages (12–15 months of age) compared to female mice (6–9 months of age). In Subramaniapillai et al. (2021), among adult humans with family history of Alzheimer's disease and APOE4 genetic risk, women appear to have more advanced brain aging than men. Nevertheless, the DL-based method used here, allowed the detection of age-related changes in the retina of younger male mice, suggesting that our approach is a powerful tool in predicting age-associated effects at earlier time points.

Age modeling alone showed statistically significant differences between 3× Tg-AD and WT mice 8 months old or older, even though the model was never trained with the two classes. It is possible that a dedicated approach could successfully distinguish the two strains, perhaps even at an earlier stage. Indeed, in Grimaldi et al. (2018), changes were seen as soon as 1–2 months after birth. The OCT is sensitive to very slight refractive index alterations along the light path. Thus, any changes in retinal content and structure ultimately influence the refractive index of the tissue, leading to changes in captured scans. If these findings are realized and verified in humans, OCT could become a powerful screening tool for AD.

The “black-box” nature of DL implies that one cannot infer the reasoning of each classification decision. For reference, we did apply deep Taylor decomposition to break down the final decision into individual contributions by relevance backpropagation (Montavon et al., 2017). Results are shown in the Supplementary Material. Nevertheless, although these methods provide an important insight, they are still limited, as we are still left unaware of how the revealed patterns link with age.

Several age-dependent cellular and molecular changes have been described in mouse retinas, such as, photoreceptor mislocalization (Sugita et al., 2020) and vascular and RPE changes (Hermenean et al., 2021). Regarding metrics captured by OCT, although some studies have found age-dependent alterations in mouse retinas, such as auto-fluorescence (Ferdous et al., 2021) and scattering diversity (Gardner et al., 2020), these cannot be directly compared with our results. Therefore, we can only speculate that some healthy age-dependent neural adaptations may be altered in transgenic animals. Indeed, delayed neural development has been suggested to occur in an AD mouse model (Rusznak et al., 2016). In addition, complex interactions between inserted mutated genes and genetic background may take place, which further hampers biological interpretations. Further functional and molecular tests will be needed to understand which factors contribute to our observations, namely the alteration in the retina aging of 3× Tg-AD mice.

## Data Availability Statement

The raw data supporting the conclusions of this article will be made available by the authors upon formal and reasonable request.

## Ethics Statement

The animal study was reviewed and approved by Animal Welfare Committee of the Coimbra Institute for Clinical and Biomedical Research.

## Author Contributions

RB accounted for fund raising. RB and PG contributed to the conceptualization and study design. RB and PS contributed to the project administration. JM performed OCT scans. PG performed data analysis. PS and PG contributed to the statistical analysis. PG, PS, JM, AA, PM, MC-B, and RB performed the analysis and interpretation of data. PG, PS, JM, and RB completed an initial review and provided significant edits and additional content before review and approval by the other authors. All authors have read, commented, and approved the manuscript.

## Funding

This study was supported by The Portuguese Foundation for Science and Technology (FCT) through PTDC/EMD-EMD/28039/2017, UIDB/04950/2020, Pest-UID/NEU/04539/2019, and by FEDER-COMPETE through POCI-01-0145-FEDER-028039.

## Conflict of Interest

The authors declare that the research was conducted in the absence of any commercial or financial relationships that could be construed as a potential conflict of interest.

## Publisher's Note

All claims expressed in this article are solely those of the authors and do not necessarily represent those of their affiliated organizations, or those of the publisher, the editors and the reviewers. Any product that may be evaluated in this article, or claim that may be made by its manufacturer, is not guaranteed or endorsed by the publisher.
