# Supplementary material for: Retinal Aging in 3× Tg-AD Mice Model of Alzheimer's Disease
Source: Front Aging Neurosci. 2022 Jun 16;14:832195. doi: 10.3389/fnagi.2022.832195 (PMC9244797; doi:10.3389/fnagi.2022.832195)
Supplement: Supplementary file 1 [file Data_Sheet_1.pdf]

Supplementary Material

1 DATA

Data characterization is shown in table S1.

2 STATISTICS

Discriminate p-values for the Kolmogorov–Smirnov and Mann-Whitney test are shown in table S2. All tests were performed with the IBM SPSS 27 software.

3 EXPLAINABILITY

Given the ‘black-box’ nature of deep learning, multiple methods to improve the explainability of these models have been suggested. We applied deep Taylor decomposition to break down the final decision into individual contributions by relevance backpropagation Montavon et al. (2017). Heatmaps that represent the relative relevance of each pixel were derived.

Supplementary figure S1 shows a selection of images for each time-point and the respective heatmaps. As shown, it appears that time-point-specific patterns emerge. Between time-points 2 and 8, the focus is in the neural-retina. For time-points 1, 12, and 16, the pattern is more diffused, centered in the posterior retina expanding up to the neural-retina. Although these methods provide an important insight, they are still limited, as we are still left unaware of how these patterns link with age.

REFERENCES

Montavon G, Lapuschkin S, Binder A, Samek W, Müller KR. Explaining nonlinear classification decisions with deep taylor decomposition. *Pattern Recognition* **65** (2017) 211–222. doi:https://doi.org/10.1016/j.patcog.2016.11.008.

Table S1. Data characterization of datasets 1 and 2 (DS1 and DS2, respectively)

**DS1**					
Time-Point	# Mice	# Acquisitions	# B-scans	% Left Eye	% Wild-type
1	90	178	1770	50.8	51.2
2	89	178	1769	48.6	48.2
3	84	168	1738	48.6	47.8
4	83	166	1681	49.1	49.0
8	78	156	1577	49.5	51.2
12	75	150	1572	50.5	49.4
16	71	141	1440	51.3	52.7
**DS2**					
Time-Point	# Mice	# Acquisitions	# B-scans	% Left Eye	% Wild-type
1	23	45	426	48.4	52.1
2	24	48	518	51.7	48.3
3	23	46	482	51.0	50.6
4	23	46	507	53.3	52.2
8	22	41	451	49.0	54.0
12	20	40	421	49.2	55.3
16	20	40	388	49.0	50.3

Table S2. Detailed p-values for the Kolmogorov–Smirnov and Mann-Whitney U tests performed with the IBM SPSS 27 software.

**Kolmogorov–Smirnov**				
	Wild-Type Trained		3×Tg-AD Trained	
Time-Point	Wild-Type	3×Tg-AD	Wild-Type	3×Tg-AD
1	3.27×10^{-3}	9.93×10^{-6}	7.21×10^{-1}	2.16×10^{-4}
2	1.88×10^{-1}	1.23×10^{-2}	2.51×10^{-3}	1.02×10^{-2}
3	6.39×10^{-1}	2.87×10^{-1}	8.10×10^{-3}	2.80×10^{-3}
4	7.65×10^{-3}	8.29×10^{-2}	7.16×10^{-3}	1.52×10^{-2}
8	1.36×10^{-2}	6.21×10^{-4}	2.44×10^{-3}	5.52×10^{-4}
12	2.83×10^{-3}	1.12×10^{-3}	1.04×10^{-1}	1.73×10^{-2}
16	3.70×10^{-4}	5.02×10^{-5}	3.48×10^{-1}	1.64×10^{-2}
**Mann-Whitney U**				
Time-Point	Wild-Type Trained		3×Tg-AD Trained	
1	3.18×10^{-4}		4.15×10^{-1}	
2	5.62×10^{-2}		6.01×10^{-3}	
3	1.27×10^{-1}		3.08×10^{-3}	
4	5.83×10^{-2}		1.23×10^{-2}	
8	3.11×10^{-3}		2.54×10^{-3}	
12	1.19×10^{-3}		6.09×10^{-3}	
16	1.33×10^{-4}		4.73×10^{-2}	

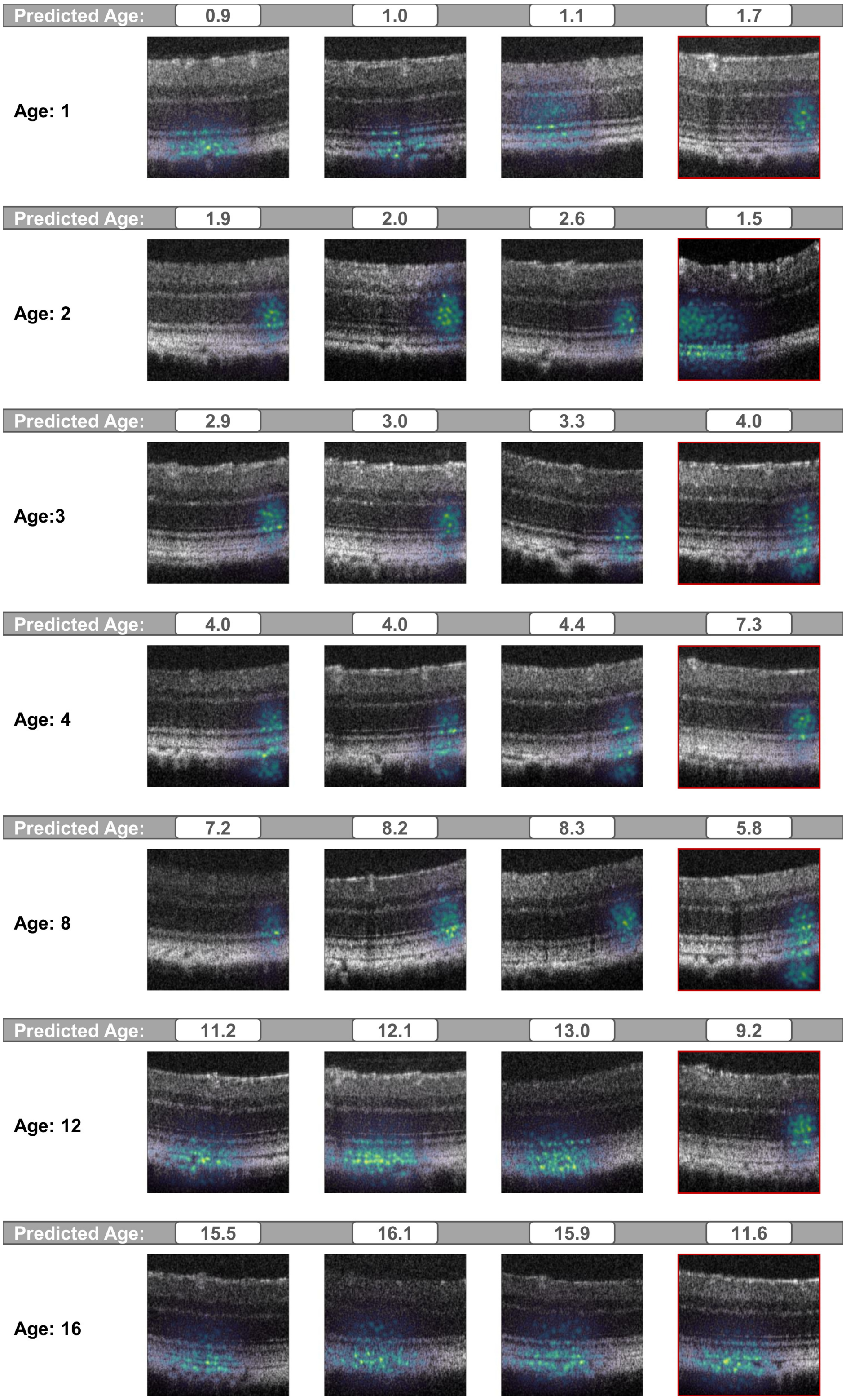

Figure S1. Selection of images for each time-point overlaid with the respective relative relevance of each pixel obtained by deep Taylor decomposition. Real age and predicted age in months are shown. All images on the right, outlined in red, are examples of considerable prediction errors.
